# Research on Precision Speed Control of TWUSM Using Discrete-Contact-Model-Based MIW-NMPC

**DOI:** 10.3390/mi17050510

**Published:** 2026-04-22

**Authors:** Yifei Guo, Kai Jing, Yuqing Wang

**Affiliations:** School of Electrical Engineering, Hebei University of Technology, Tianjin 300401, China; guoif_tz@163.com (Y.G.);

**Keywords:** traveling wave ultrasonic motor, discrete contact modeling, nonlinear model predictive control, precision speed control

## Abstract

Aiming at the problems of low control efficiency and great regulation difficulty of traveling wave ultrasonic motors (TWUSMs) caused by nonlinearity, strong coupling characteristics, and parameter uncertainty, this paper proposes a speed regulation scheme based on discrete contact modeling and monotonically increasing weighted nonlinear model predictive control (MIW-NMPC). On the basis of analyzing the existing TWUSM models, a discrete contact model of the motor is constructed, which reduces the modeling complexity while completely retaining the core dynamic characteristics of the motor’s operating mechanism. Based on this model, the MIW-NMPC algorithm for the precision speed control of TWUSM is designed, and its feasibility and stability are deduced and verified. Comparative simulations and experimental results demonstrate that the proposed discrete-model-based MIW-NMPC scheme can effectively enhance the precision and dynamic response of TWUSM speed control, offering a novel technical approach for its high-precision speed regulation applications.

## 1. Introduction

A traveling wave ultrasonic motor (TWUSM) is a special type of motor featuring the advantages of low speed, high torque, immunity to magnetic field interference, flexible structural design, and excellent position and speed control performance [1]. It is thus widely applied in the high-precision motion control field, including the medical, aerospace, and weapon guidance sectors [2,3]. However, the TWUSM’s control efficiency and accuracy are severely constrained by its inherent strong nonlinearity, severe coupling characteristics, and parameter uncertainty, arising from the intricate operating mechanism of its inverse piezoelectric effect and vibrational friction.

To address the above control challenges, existing TWUSM control research is mainly categorized into model-based and model-free approaches. For model-based methods, most designs rely on simplified mechanistic models (e.g., approximate time-domain models, Hammerstein models) to develop controllers such as adaptive sliding mode [4], backstepping [5], and iterative learning control [6,7,8], which offer physical interpretability but lose the core discrete stator–rotor contact mechanism due to model simplification, resulting in inherent limitations in modeling fidelity. For model-free methods, data-driven techniques including neural networks [9], deep reinforcement learning [10], and extremum seeking [11] are employed to achieve robust control, which eliminates the need for explicit modeling but lack physical interpretability, suffering from high parameter tuning difficulty and insufficient dynamic performance optimization. Overall, existing methods are insufficient to strike a desirable balance between accurate modeling that retains the main complex nonlinear dynamic characteristics of TWUSMs and the design of robust controllers with enhanced performance.

To remedy this deficiency for TWUSMs, an improved NMPC scheme based on a new discrete contact model is studied to balance model fidelity and control performance, and no related work on TWUSMs has been reported. Based on the core requirements of TWUSM control, Table 1 compares and analyzes the comprehensive performance of typical NMPC methods in recent years from the key dimensions including terminal constraints, stability proof, computational cost, adaptability to nonlinearity, and embedded real-time performance.

According to the multi-index comprehensive evaluation, the monotonically increasing weighted NMPC (MIW-NMPC) shows better overall performance and is more suitable for the practical control of TWUSMs. Furthermore, to realize the practical application of MIW-NMPC in high-precision speed control of TWUSMs, a discrete TWUSM model is also proposed by fully capturing its operating mechanism.

This paper is structured as follows: Section 2 proposes and verifies a discrete contact torque model for TWUSMs through the analysis of traditional models. Section 3 designs the MIW-NMPC algorithm with the discrete contact torque model and proves its stability. Section 4 evaluates the algorithm via comparative simulations and experiments, and Section 5 summarizes the research results and draws relevant conclusions.

## 2. Discrete Model of Ultrasonic Motor

### 2.1. TWUSM Basic Model

According to the TWUSM stator electromechanical conversion and stator–rotor contact action operation mechanism. The model can be described by the electromechanical coupling equation, current equation, traveling wave motion equation, torque equation, and driving equation:

(1) $$\left\{\begin{array}{l} M_{s}\ddot{w}+D_{s}\dot{w}+C_{s}w=\Theta u+F_{c}\\ i=R_{d}^{-1}u+C_{d}\dot{u}+\Theta \dot{w}\\ w(t)=w_{A}\cos k\theta +w_{B}\sin k\theta =W\cos\left(k\theta -k\theta_{C}\right)\\ T_{e}=\xi (W(k),\omega_{R}(k)),\;W(k)\in G,\omega_{R}(k)\in N\\ J_{R}\frac{d\omega_{R}}{dt}=T_{e}-T_{load} \end{array}\right.$$

where Ms=msA  msB is the two-phase modal mass matrix; ***D***_s_ and ***C***_s_ are the two-phase modal damping matrix and modal stiffness matrix, respectively; ***R***_d_ and ***C***_d_ are the dielectric loss resistance and static capacitance resistance matrices of the two-phase ceramics, respectively, both of the same type as ***M***_s_; $w={\left[\begin{array}{cc} w_{A}, & \left.w_{B}\right] \end{array}\right.}^{T}$ is the two-phase vibration mode; $u={\left[\begin{array}{cc} u_{A}, & u_{B} \end{array}\right]}^{T}$ is the two-phase excitation voltage; $i={\left[\begin{array}{cc} i_{A}, & i_{B} \end{array}\right]}^{T}$ is the two-phase current; *J*_R_ is the rotational inertia; *ω*_R_ is the rotational angular velocity; *T*_load_ is the load torque; *W* denotes the amplitude of the traveling wave; and *G* and *N* represent the range of input vibration modes and the operating speed range of the rotor, respectively.

When the two-phase vibration modes are of equal amplitude and are orthogonal, they can be expressed as:

(2) $$w(t)=W_{0}\cos(k\theta -\omega t)$$

The torque equation in (1) is the core of converting the traveling wave vibrations into contact friction for the TWUSM. Strong nonlinearities in the process make control difficult. For the torque expression, the current studies contain the following four types.

• Spatial distribution-based torque calculation [19,20,21]:

Relying on the spatial force distribution of traditional analytical models, the torque is described as the contact friction generated on the contact area by the relative motion between the stator and rotor and excited by the traveling wave, which is capable of revealing the internal mechanism yet is complex in calculation.

• Time scale-based torque calculation [22,23,24]:

By using single-particle periodic motion to analyze stick–slip friction between the stator and rotor, the torque is obtained within one traveling wave period, which, like the above model, relies on traditional analytical models.

• Mass motion-based torque calculation [25,26]:

The stator–rotor interaction is simplified into a lumped linear mass–spring–damper system. A point-contact mechanism that considers a single contact point per vibration wavelength is adopted for torque calculation, which cannot fully characterize the spatial distribution of contact forces across the contact zone.

• Nonlinear identification-based torque calculation [27,28]:

Data-driven models (neural networks, state-space, nonlinear ARX, Hammerstein-Wiener, etc.) estimate torque through parameter identification, which cannot reveal the physical mechanism of stator–rotor contact.

In summary, the first two models are commonly used to reveal the operating mechanism of the motor, but they involve complex computations and are rarely adopted for control purposes. The latter two models are simpler than the former yet cannot characterize the stator–rotor contact interaction. In particular, the fourth model depends entirely on the motor’s operational data. Therefore, this paper proposes a new discrete modeling approach that is both computationally efficient and capable of accurately describing the physical mechanism.

### 2.2. Torque Calculation Based on Discrete Contact Model

To simplify the torque calculation procedure in the dynamic mechanism model, a discrete contact model is proposed. The stator surface within one wavelength is discretized into *n* equally spaced points, which are denoted as *kθ*^(^*^j^*^)^ in the rotating coordinate system of the traveling wave crest, and are given as:

(3) $$\begin{array}{c} k\theta^{\left(j\right)}=j\frac{2\pi }{n}\;\left(j=0,\pm 1,\pm 2,\cdots \pm \left(\frac{n}{2}-1\right),\frac{n}{2}\right) \end{array}$$

where *k* is the wave number.

1. Motion equation of discrete points:

By examining the contact neighborhood of the discrete point, the average axial displacement and circumferential velocity can be expressed as:

(4) $$u_{\mathrm{sz}}^{\left(j\right)}=\phi_{z}W\frac{n}{2\pi }\int_{k\theta^{\left(j\right)}-\frac{\pi }{n}}^{k\theta^{\left(j\right)}+\frac{\pi }{n}}\cos k\theta dk\theta =\phi_{z}W\frac{\sin\pi /n}{\pi /n}\cos k\theta^{\left(j\right)}$$

(5) $$v_{s\theta }^{\left(j\right)}=-\phi_{\theta }V\frac{n}{2\pi }\int_{k\theta^{\left(j\right)}-\frac{\pi }{n}}^{k\theta^{\left(j\right)}+\frac{\pi }{n}}\sin\left(k\theta +k\theta_{T}\right)dk\theta =-\phi_{\theta }V\frac{\sin\pi /n}{\pi /n}\sin(k\theta^{\left(j\right)}+k\theta_{T})$$

where *ϕ*_z_ and *ϕ*_θ_ denote the mode shape coefficients in the radial and circumferential directions, respectively; and $W=\sqrt{w_{A}^{2}+w_{B}^{2}}$, $V=\sqrt{{\dot{w}}_{A}^{2}+{\dot{w}}_{B}^{2}}$. Under pure traveling wave conditions, *kθ*_T_ is approximated as −π/2.

2. Force equation of discrete points:

The motion of discrete points in the axial and circumferential directions exerts forces on the rotor, which can be expressed as symmetrically distributed axial contact pressure and circumferential friction under pure traveling wave conditions, which are deduced by sum-to-product identities:

(6) $$\begin{array}{ll} p^{\left(j\right)} & =c_{N}(u_{\mathrm{sz}}^{\left(j\right)}-u_{\mathrm{sz}}^{\left(m\right)})\\ & =2c_{N}\phi_{z}W\frac{\sin\pi /n}{\pi /n}\left[\sin\frac{(m+j)\pi }{n}\sin\frac{(m-j)\pi }{n}\right]\;\left|j\right|\leq m \end{array}$$

(7) fθj=sgnvsθj−vRμpj=sgnl−jμpj j≤m

where *c*_N_ is the linear equivalent stiffness of the friction layer; ±m are the contact boundary indices of the discrete points where *p*^(±m)^ = 0; *μ* denotes the friction coefficient; and ±*l* are the iso-velocity indices of the discrete points that the discrete friction exhibits both driving and resisting characteristics within the contact region.

3. Torque equation of TWUSMs:

Based on the distribution of the frictional force, the total torque can be expressed as:

(8) $$T=2r\mu \sum_{j=-l}^{l}p^{\left(j\right)}-r\mu \sum_{j=-m}^{m}p^{\left(j\right)}$$

where *m* is determined by the Newton–Raphson iteration using the axial force equilibrium equation.

(9) $$\begin{array}{ll} F_{\mathrm{pre}} & \approx F_{N}=\sum_{j=-m}^{m}p^{\left(j\right)}\\ & =2c_{N}\phi_{z}W\frac{n}{2\pi }\left[\left(2m+1\right)\sin\left(\frac{\left(2m-1\right)\pi }{n}\right)+\left(2m-1\right)\sin\left(\frac{\left(2m+1\right)\pi }{n}\right)\right] \end{array}$$

where *F*_pre_ represents the rotor preload force, and *F*_N_ denotes the total axial pressure exerted by the stator on the rotor. For the equation *g*(*m*) = *F*_N_ − *F*_pre_ = 0, the Newton–Raphson iteration scheme is given by:

(10) $$m_{q+1}=m_{q}-\frac{g\left(m_{q}\right)}{g^{\prime }\left(m_{q}\right)}$$

where *q* is the iteration index.

Clearly, function *g*(*m*) is continuously differentiable in the physically feasible domain 0 ≤ *m* < *n*, and its derivative *g*′(*m*) is non-zero everywhere, satisfying the local quadratic convergence condition of the Newton–Raphson method. With a physically reasonable initial guess (e.g., *m*_0_ = 0 or *n*/2), the iteration converges to the unique solution within 3–5 steps without divergence. Ultimately, rounding the iterative result to the nearest integer yields the value of *m*.

Thus, the discrete torque calculation method for the TWUSM is developed. As *n* → ∞, the proposed discrete model reduces to the continuous spatial distribution model. In contrast with identification models, the proposed model preserves the physical contact mechanism; compared with mass–spring models, it can characterize the force distribution over the contact zone.

**Figure 1:**
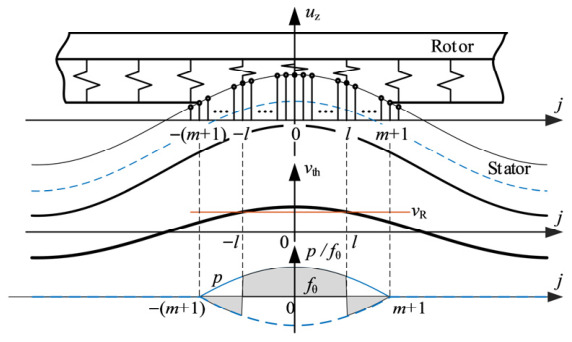
Distribution of discrete points, motion, and force.

The discrete points, motion, and force distribution in the above analysis are shown in Figure 1.

**Figure 2:**
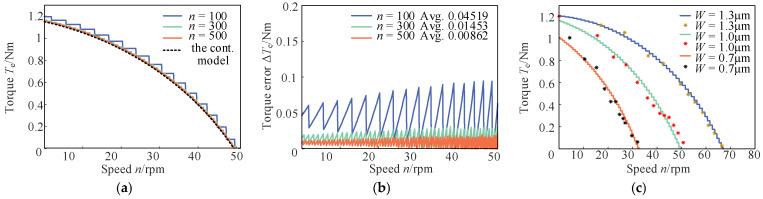
Speed–torque curves of TWUSMs under discrete modeling. (**a**) Speed–torque characteristics under different discretization numbers; (**b**) Torque modeling errors under different discretization numbers; (**c**) Speed–torque characteristics under different vibration mode amplitudes.

Based on the practical parameters of the TWUSM (*k* = 9, *r* = 27.58 mm, *μ* = 0.2, etc.), the speed–torque curves of the discrete model under *W* = 1 μm are plotted in Figure 2a. As the discretization number *n* increases, the curve becomes smoother and gradually converges to that of the continuous spatial distribution model depicted by dashed line. The torque errors are presented in Figure 2b, where the modeling error decreases significantly as *n* grows. Meanwhile, the proposed discrete model exhibits a linear computational complexity of *O*(*n*), implying that the computational load increases linearly with *n*. Therefore, a favorable trade-off between modeling accuracy and computational efficiency can be achieved by appropriately selecting the discretization number *n*.

Furthermore, Figure 2c presents the speed–torque curves versus modal vibration amplitude W at *n* = 300. The measured characteristics of the actual motor under identical W are marked by asterisks, showing excellent agreement with the model curves. This result further validates the rationality of the proposed model in this paper.

## 3. Discrete-Model-Based NMPC for Precision Speed Control of TWUSM

### 3.1. Design of MIW-NMPC for TWUSM Speed Control

Based on the discrete torque model, a monotonically increasing weighted nonlinear model predictive control (MIW-NMPC) scheme without terminal constraints is designed for the high-precision speed control of TWUSMs. Under pure traveling wave conditions, the torque can be expressed as a function of the vibration mode amplitude *W* and rotor speed *ω*_R_.

(11) $$T_{e}(k)=\xi (W(k),\omega_{R}(k)),\;W(k)\in G,\omega_{R}(k)\in N$$

From the motion function given in Equation (1), we let the input variable *u* = *W* and the state variable *x* = *ω*_R_ − *ω*_d_ (where *ω*_d_ denotes the target angular velocity). The state equation discretized with sampling period *t*_s_ can be expressed as:

(12) $$x(k+1)=x(k)+\frac{t_{s}}{J_{R}}\left(T_{e}-T_{\mathrm{load}}\right)=x(k)+\frac{t_{s}}{J_{R}}\left[\xi \left(u\left(k\right),x\left(k\right)+\omega_{d}\right)-T_{\mathrm{load}}\right]$$

Considering the control sequence ***u*** = [*u*_0_, *u*_1_, …, *u*_N−1_] over the prediction horizon of length *N*, the predictive state trajectory can be expressed with *i* = 0, 1, …, *N*:

(13) $$\begin{array}{l} x_{0}^{u}\left(k\right)=x\left(k\right)\\ x_{i}^{u}\left(k\right)=x_{i-1}^{u}\left(k\right)+\frac{t_{s}}{J_{R}}\left[\xi \left(u_{i-1}\left(k\right),x_{i-1}^{u}(x)+\omega_{d}\right)-T_{\mathrm{load}}\right] \end{array}$$

The cost function of MIW-NMPC can be expressed as follows:

(14) $$\begin{array}{c} J_{m}=\sum_{i=1}^{N}w_{i}x_{i}^{u}{\left(k\right)}^{T}Qx_{i}^{u}\left(k\right)=\sum_{i=1}^{N}{\left(\frac{i}{N}\right)}^{m}Qx_{i}^{u}{\left(k\right)}^{2}\;s.t.\;x_{\min}^{u}\leq x_{i}^{u}\leq x_{\max}^{u}. \end{array}$$

where *Q* are one-dimensional positive constant matrices (i.e., scalars), since the state $x_{i}^{u}$ belong to ℝ.

The optimal control input u*(*k*) is obtained by minimizing the cost function via receding horizon optimization using particle swarm optimization (PSO). This approach fully leverages the core advantages of PSO—independence from objective function gradients, fast convergence speed, simple parameter tuning, strong global search capability, and excellent adaptability to nonlinear and non-convex problems—thus overcoming the local optimality limitation of traditional gradient-based methods and enabling precise speed control. The optimization process is detailed in the pseudocode of Table 2.

To enhance the disturbance rejection performance of the system under load variations, a PI-type load torque observer is designed for the TWUSM. The torque estimation law is given by:

(15) $${\hat{T}}_{L}=\left(K_{p}+\frac{K_{i}}{s}\right)\left(\omega^{*}-\omega \right)$$

where ω* is the reference speed, *ω* is the measured speed, and *K*_p_ and *K*_i_ are the observer gains, which can achieve fast and accurate load torque estimation. By feeding the estimated load torque forward into the control loop, the influence of load disturbances can be significantly attenuated, and the robustness and steady-state control accuracy of the system can be further improved.

For achieving pure traveling wave vibration, the nonlinear SMO and coordination controller from our previous study [29] are used to estimate and control vibration modes coordinately.

### 3.2. Stability Proof of MIW-NMPC

For nonlinear model predictive control, stability is a critical issue. It is necessary to analyze the stability conditions of the control system to guarantee a stable closed-loop system.

We suppose that at time *k*, an optimal control sequence $u^{*}=\left(u_{0}^{*},u_{1}^{*},\cdots ,u_{N-1}^{*}\right)\in U$ exists that minimizes the monotonically increasing weighted cost function J^*^_m_. Then,

(16) $$J_{m}^{*}=\min J_{m}\left(\left.u^{*}\right|x\left(k\right)\right)=\sum_{i=1}^{N}{\left(\frac{i}{N}\right)}^{m}Qx_{i}^{u^{*}}{\left(k\right)}^{2}$$

We let the optimal control sequence at time *k* + 1 be $\tilde{u}=\left(u_{1}^{*},u_{2}^{*},\ldots u_{N-1}^{*},\bar{u}\right)$, where

(17) $$\bar{u}=\arg\underset{u\in U}{\min}\;Qx_{N+1}^{u^{*}}{\left(k\right)}^{2}$$

Then, the costliest function at the moment *k* + 1 is:

(18) $$\begin{array}{ll} J_{m}\left(\left.\tilde{u}\right|x_{k+1}\right) & =\sum_{j=2}^{N}{\left(\frac{j-1}{N}\right)}^{m}Qx_{j}^{u^{*}}{\left(k\right)}^{2}+Qx_{N}^{\bar{u}}{\left(k+1\right)}^{2}\\ & =J_{m}^{*}-\frac{1}{N^{m}}Qx_{1}^{u^{*}}{\left(k\right)}^{2}-\sum_{j=2}^{N}\left[1-{\left(\frac{j-1}{j}\right)}^{m}\right]{\left(\frac{j}{N}\right)}^{m}Qx_{j}^{u^{*}}{\left(k\right)}^{2}+Qx_{N}^{\bar{u}}{\left(k+1\right)}^{2} \end{array}$$

and since for all *j* ∈ {2, …, *N*}, one has $\left[1-{\left(\frac{j-1}{j}\right)}^{m}\right]\geq \left[1-{\left(\frac{N-1}{N}\right)}^{m}\right]$

(19) $$J_{m}\left(\left.\tilde{u}\right|x_{k+1}\right)\leq J_{m}^{*}-\frac{1}{N^{m}}Qx_{1}^{u^{*}}{\left(k\right)}^{2}-\left[1-{\left(\frac{N-1}{N}\right)}^{m}\right]\sum_{j=2}^{N}{\left(\frac{j}{N}\right)}^{m}Qx_{j}^{u^{*}}{\left(k\right)}^{2}+Qx_{N}^{\bar{u}}{\left(k+1\right)}^{2}$$

and keeping only the last term of the sum in the first term, we obtain:

(20) $$\begin{array}{ll} J_{m}\left(\left.\tilde{u}\right|x_{k+1}\right) & \leq J_{m}^{*}-\frac{1}{N^{m}}Qx_{1}^{u^{*}}{\left(k\right)}^{2}-\left[1-{\left(\frac{N-1}{N}\right)}^{m}\right]Qx_{N}^{u^{*}}{\left(k\right)}^{2}+Qx_{N}^{\bar{u}}{\left(k+1\right)}^{2}\\ & \leq J_{m}^{*}-\frac{1}{N^{m}}Qx_{1}^{u^{*}}{\left(k\right)}^{2}+{\left(\frac{N-1}{N}\right)}^{m}Qx_{N}^{u^{*}}{\left(k\right)}^{2}+Q\left[x_{N}^{\bar{u}}{\left(k+1\right)}^{2}-x_{N}^{u^{*}}{\left(k\right)}^{2}\right] \end{array}$$

By virtue of the local control invariance property, we have:

(21) $$\begin{array}{cc} Qx^{2}\left(k+1\right)-Qx^{2}\left(k\right)\leq -\gamma Qx^{2}\left(k\right), & \gamma >0 \end{array}$$

So,

(22) $$J_{m}\left(\left.\tilde{u}\right|x_{k+1}\right)\leq J_{m}^{*}-\frac{1}{N^{m}}Qx_{1}^{u^{*}}{\left(k\right)}^{2}-\left[\gamma -{\left(\frac{N-1}{N}\right)}^{m}\right]Qx_{N}^{u^{*}}{\left(k\right)}^{2}$$

It suffices to satisfy the condition of $\gamma \geq {\left(\frac{N-1}{N}\right)}^{m}$ to guarantee convergence, in which ${\left(\frac{N-1}{N}\right)}^{m}\to 0$ as m → ∞. The stability of the MIW-NMPC is thus established.

## 4. Simulations and Experiments

### 4.1. Analysis of Control Parameters for MIW-NMPC

For the parameter design and the convergence of the discrete-contact-model-based MIW-NMPC, the weighting parameter *Q* is empirically selected in the range of 50–100, and simulation results show that it has little influence on the algorithm performance.

**Figure 3:**
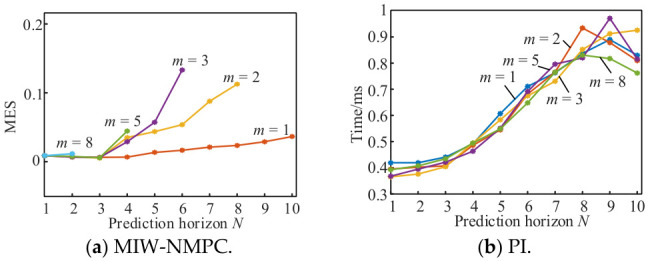
Simulation results of convergence and parameter selection.

The prediction horizon *N* and the weighting exponent *m* are analyzed through multiple speed tracking simulations under different reference velocities. The comprehensive mean squared error (MSE) and average single-step computation time are shown in Figure 3a, where only results with MSE < 0.2 are presented. It can be observed that small MSE values are achieved for *m* ≤ 3 over the effective range of N, with the minimum MSE of 0.005362 obtained at *m* = 3 and *N* = 3. The single-step computation time is similar for different values of *m* and increases with *N* in Figure 3b; for *m* ≤ 3, the computation time remains below 0.5 ms. Accordingly, *Q* = 80, *m* = 3, and *N* = 3 are adopted for simulation verification in this paper.

For a fair comparison, the conventional MPC in the control group is configured with the same *Q*, *N*, and PSO scheme. Furthermore, a practical PI controller is also adopted as a comparative scheme, with its proportional gain *K*_p_ and integral gain *K*_i_ set to 0.004 and 0.0001, respectively.

### 4.2. Simulations of TWUSM Precise Speed Control Using MIW-NMPC

In this part, simulation verification of the MIW-NMPC speed control scheme for the TWUSM is carried out. Four operating conditions are adopted to compare and validate the proposed method with conventional MPC and PI control.

**Figure 4:**
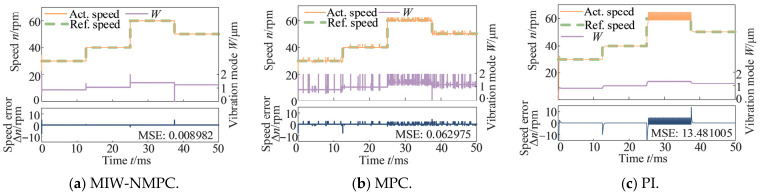
Control results under steps speed reference with a constant load torque.

Figure 4 presents the control results under steps speed reference with a constant load torque of 0.5 N·m. It can be observed from the figure that the actual speed of the MIW-NMPC method can track the reference speed accurately, and the control input (vibration mode *W*) varies stably. Although the conventional MPC can also achieve speed tracking, it exhibits obvious fluctuations in the speed response, and the control input W has significant fluctuations. For the PI control, the speed response shows overshoot, lag, and chattering at high speeds. The speed deviations of the three methods evaluated by the MSE are 0.008982 (MIW-NMPC), 0.062975 (MPC), and 13.481005 (PI).

**Figure 5:**
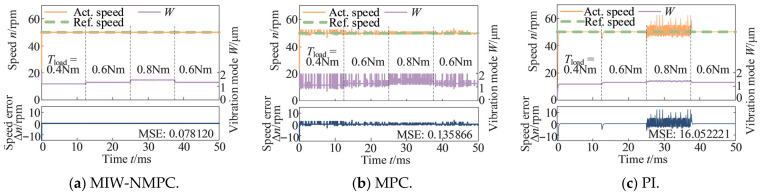
Control results under constant speed reference with steps load torque.

Figure 5 sets a constant reference speed of 50 rpm with step-changed load torque to reflect the disturbance rejection capability of the control algorithms. It can be seen from the figure that when the load torque changes, the control input W of the MIW-NMPC can respond quickly to cope with the load variation, thereby stabilizing the speed, while the other two methods have obvious speed fluctuations when the load changes. The MSE values of the speed deviations are 0.078120 (MIW-NMPC), 0.135866 (MPC), and 16.052221 (PI).

**Figure 6:**
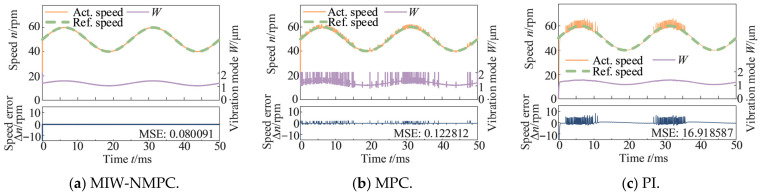
Control results under sinusoidal speed reference with a constant load torque.

Figure 6 shows the control results under a sinusoidal speed reference around 50 rpm with a constant load torque of 0.5 N·m. It can be observed that the proposed algorithm can track the speed variation well, and the control input changes smoothly. The MSE values of the speed deviations are 0.080091 (MIW-NMPC), 0.122812 (MPC), and 16.918587 (PI).

**Figure 7:**
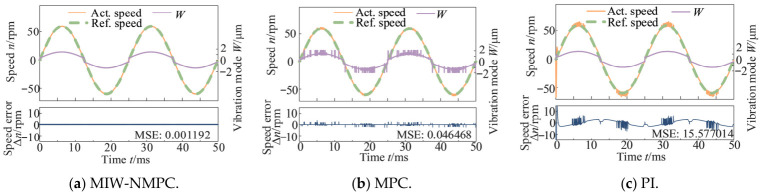
Control results under forward and reverse sinusoidal speed reference.

Figure 7 presents the control results under forward and reverse sinusoidal speed reference, demonstrating that the proposed algorithm yields superior performance in bidirectional speed regulation. The MSE values of the speed deviations are 0.001192 (MIW-NMPC), 0.046468 (MPC), and 15.577014 (PI).

Overall, the above simulations demonstrate that the proposed algorithm has obvious advantages in speed tracking, which can perform fast and stable regulation to track the given speed and resist load disturbances.

### 4.3. Experimental Validation

**Figure 8:**
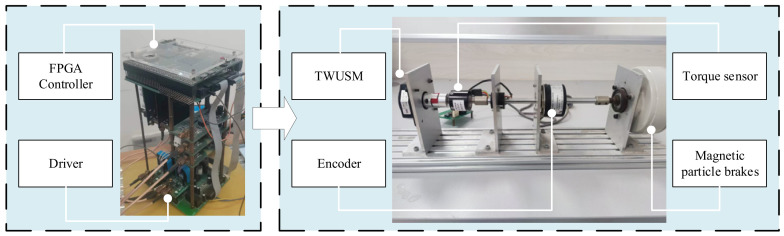
ARM-FPGA-based TWUSM experimental platform.

To verify the practical performance of the proposed MIW-NMPC algorithm, an FPGA-ARM-based control platform for the TWUSM is established in this study, as shown in Figure 8, and the key parameters of the platform are listed in Table 3.

**Figure 9:**
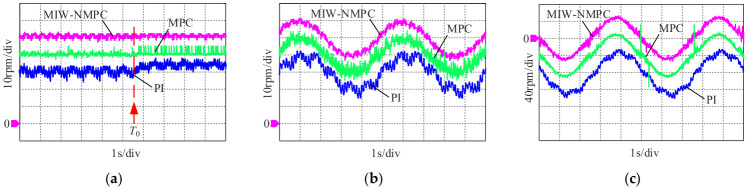
Experiment results. (**a**) Steady-state speed disturbance rejection performance; (**b**) Sinusoidal speed tracking performance; (**c**) Bidirectional sinusoidal speed tracking performance.

Experiments are carried out on the above platform for a precision feed system. For algorithm comparison and verification, the control frequency of both the proposed MIW-NMPC and the comparative MPC algorithm is uniformly set to 2 kHz in this paper, and FPGA is adopted to implement the parallel acceleration of PSO rolling optimization to improve the online computing speed. Meanwhile, the PID algorithm for real-time control is also employed for comparison, with its control frequency set to 41 kHz. The experiments are conducted under three typical working conditions: sudden load torque change, variable speed operation, and forward/reverse rotation control. The experimental results are shown in Figure 9, where all comparative curves are shifted downward by 1 and 2 divisions for better visualization.

In Figure 9, the horizontal axis is time with a scale of 1 s/div, and the vertical axis is speed with scales of 10 rpm/div for (a) and (b), and 40 rpm/div for (c). Specifically, Figure 9a shows the steady-state performance when the brake load is suddenly reduced from 3 Nm to 0 at time *T*_0_, where the proposed algorithm achieves a speed fluctuation amplitude of less than 3 rpm, only half of that of the conventional MPC, and less than 1/3 of that of the PI controller, demonstrating the optimal steady-state disturbance rejection capability.

Figure 9b depicts the speed control under a sinusoidal reference speed with a constant load torque, where the tracking error amplitude of the proposed algorithm is less than 5 rpm, only about 50% of that of the conventional MPC and 20–30% of that of the PI, while the PI exhibits significant phase lag.

Figure 9c shows the bidirectional sinusoidal speed tracking performance. The proposed method achieves a tracking error of less than 8 rpm, which is significantly smaller than that of the conventional MPC and the PI controller, of which the latter still has phase lag.

Experimental results demonstrate that the proposed algorithm can effectively improve the dynamic response and control precision of the TWUSM speed control system.

## 5. Conclusions

To address the strong nonlinearity and electromechanical coupling of TWUSMs, this paper proposes a discrete-contact-model-based MIW-NMPC high-precision speed control strategy. The discrete model simplifies torque calculation while retaining the motor’s core dynamic characteristics, providing an accurate foundation for algorithm design. Based on this model, the MIW-NMPC algorithm is designed, and its stability is rigorously proven. Comparative simulations and experiments with traditional MPC and PI control algorithms show that the proposed strategy exhibits excellent performance in speed regulation precision and dynamic response speed. This research offers a novel feasible control scheme for high-precision TWUSM speed regulation and provides a reference for the control of other nonlinear and strong-coupling electromechanical systems.

## Figures and Tables

**Table 1:** Comparison of typical NMPC methods for TWUSM control.

Method	Terminal Constraints	Stability Proof	Computational Cost	Adaptability to Nonlinearity	Embedded Real-Time Performance	Suitability for TWUSMs
Traditional NMPC [12]	Required	Complex	Very high	Weak	Very poor	Unsuitable
Tube-Robust NMPC [13]	Required	Complex	High	Medium	Poor	Unsuitable
Learning-Based NMPC [14]	Not required	None	High	Strong	General	Limited
Contraction NMPC [15]	Not required	Rigorous	Medium	Medium	General	Medium
Model-Free NMPC [16]	Not required	None	Medium	Medium	General	Medium
MIW-NMPC [17,18]	No	Rigorous	Medium	Strong	Good	Optimal

**Table 2:** PSO-based NMPC.

Input: Current state *x*(*k*), reference trajectory, constraints. Output: Optimal control input u*(*k*).
Initialize swarm: particle positions *X*, velocities *V*; *P*_best_ = X, *G*_best_ = arg min *J*(*X*). While termination criteria not met: Evaluate cost *J* for each particle; Update *P*_best_ (individual best) and *G*_best_ (global best); Update particle velocity: *v_i_* = *ωv_i_* + *c*_1_*r*_1_(*P*_best,_*_i_* − *x_i_*) + *c*_2_*r*_2_(*G*_best_ − *x_i_*); Update particle position: *x_i_* = *x_i_* + *v_i_* (subject to constraints). 3. Set ***x***_opt_ = *G*_best_ from final swarm. 4. Return ***x***_opt_(1) as u*(*k*).

**Table 3:** Experimental platform parameters.

Parameter	Specification	Parameter	Specification
ARM Chip	GD32F450	TWUSM Type	TRUM-60
FPGA Chip	SL2S-25E-8U213C	TWUSM Speed	60 rpm (max. 150 rpm)
Control Frequency	2 kHz	TWUSM Torque	0.6 Nm (max 1.2 Nm)
Driver	Dual full-bridge resonant driver	TWUSM Operating Frequency	40–44 kHz
Driver Output Voltage	0–200 VAC	Magnetic Powder Brake	0–3 Nm (24 V)
Encoder Resolution	2500 lines/rev	Torque Sensor Sensitivity	2.9 mV/V (0–5 Nm)

Note: ARM Chip: GD32F450, GigaDevice Semiconductor Inc., Beijing, China FPGA Chip: SL2S-25E-8U213C, Lattice Semiconductor, Shanghai, China TWUSM Type: TRUM-60, Chunsheng Ultrasonic Motor Co., Ltd., Nanjing, Jiangsu, China.

## Data Availability

The original contributions presented in this study are included in the article. Further inquiries can be directed to the corresponding author.
